# Survival to Parasitoids in an Insect Hosting Defensive Symbionts: A Multivariate Approach to Polymorphic Traits Affecting Host Use by Its Natural Enemy

**DOI:** 10.1371/journal.pone.0060708

**Published:** 2013-04-02

**Authors:** Emilie Bilodeau, Jean-Frédéric Guay, Julie Turgeon, Conrad Cloutier

**Affiliations:** Département de Biologie, Université Laval, Québec, Québec, Canada; French National Institute for Agricultural Research (INRA), France

## Abstract

Insect parasitoids and their insect hosts represent a wide range of parasitic trophic relations that can be used to understand the evolution of biotic diversity on earth. Testing theories of coevolution between hosts and parasites is based on factors directly involved in host susceptibility and parasitoid virulence. We used controlled encounters with potential hosts of the *Aphidius ervi* wasp to elucidate behavioral and other phenotypic traits of host *Acyrthosiphon pisum* that most contribute to success or failure of parasitism. The host aphid is at an advanced stage of specialization on different crop plants, and exhibits intra-population polymorphism for traits of parasitoid avoidance and resistance based on clonal variation of color morph and anti-parasitoid bacterial symbionts. Randomly selected aphid clones from alfalfa and clover were matched in 5 minute encounters with wasps of two parasitoid lineages deriving from hosts of each plant biotype in a replicated transplant experimental design. In addition to crop plant affiliation (alfalfa, clover), aphid clones were characterized for color morph (green, pink), *Hamiltonella defensa* and *Regiella insecticola* symbionts, and frequently used behaviors in encounters with *A. ervi* wasps. A total of 12 explanatory variables were examined using redundancy analysis (RDA) to predict host survival or failure to *A. ervi* parasitism. Aphid color was the best univariate predictor, but was poorly predictive in the RDA model. In contrast, aphid host plant and symbionts were not significant univariate predictors, but significant predictors in the multivariate model. Aphid susceptibility to wasp acceptance as reflected in host attacks and oviposition clearly differed from its suitability to parasitism and progeny development. Parasitoid progeny were three times more likely to survive on clover than alfalfa host aphids, which was compensated by behaviorally adjusting eggs invested per host. Strong variation of the predictive power of intrinsic (body color) and extrinsic traits (symbionts, host plant), indicate that host variables considered as key predictors of outcomes strongly interact and cannot be considered in isolation.

## Introduction

Parasitoids are insects with indirect development (metamorphosis) whose immature stages are parasitic of other insects [1], [2], [3], [4]. They differ from true parasites in causing high parasitic stress and harm and invariably killing their host. The parasitoid larva is a secondarily evolved and often ‘brutal’ parasite (sections 1.5.1 & 8.3.3 in [5]; p. 9 in [6]), which may even force its host to participate in its own death [7]. In contrast to the larval stage, the adult parasitoid is an independent free-living and flying insect (in most cases a wasp) expressing complex behavior [3], [5], [6], [8], [9]. The adult parasitoid is responsible for transmission to new hosts at each generation, an essential feature of parasitoid life, generally excluding within-host multiplication and thus setting them apart from microbial parasites or pathogens [10], [11].

Most parasitoids exploit their hosts as endoparasitic koinobionts [3], [5], [6], [12], [13], [14]. While elaborate physiological and molecular adaptations are required of the parasitic stages to face host resistance and immunity [3], high mobility and elaborate behavior are required of the parasitoid adult wasp to find, recognize, and physically break defenses of appropriate hosts, to transmit progeny able to survive in the host (although wasp venom factors can be important).

A parasitoid can cause significant mortality and exert strong selection pressures for resistance in a variable host population, potentially leading in turn to evolutionary responses on the parasitoid side [15], [16], [17]. This could explain why most parasitoids specialize on a limited number of hosts [5], [6]. As in other forms of specialization [18], a parasitoid wasp genotype should recognize and/or be able to establish in only a subset of available host genotypes in genetically variable populations (reviewed in [19]). Similarly, a host genotype should be able to avoid, resist or sustain parasites of only a subset of all potential challengers. A central question is thus whether and how coevolution between parasitoids and their hosts determine parasitoid specialization in space and time [5], [20], [21].

Theories of coevolution predict that reciprocal selection pressures in space and time is reflected in polymorphism of host and parasite traits [22], [23], [24], [25], [26]. Testing such theories requires good knowledge of factors directly involved in host susceptibility and parasitoid virulence, which are still poorly documented except in a few insect systems such as those based on *Drosophila* hosts ([16]; reviewed in [27]).

The traits that determine if potential protagonists can establish a parasitic relation are studied under the mirror concepts of parasite virulence vs. host resistance [17]. For an insect parasitoid, virulence is expressed sequentially by two forms of strongly divergent lifestyles, first behaviorally at the step of transmission by the wasp, and then physiologically (or parasitically) at that of host exploitation by the larva. Host preference ([28] and references therein) refers to the adult behavior traits of virulence, which determine recognition and orientation to hosts, and expression of motor patterns to defeat host behavioral and physical defenses. Virulence traits of the parasitic stages (eggs and early larval stages, and associated venom injected by the wasp, reviewed in [3]) interact directly with host resistance and immunity.

This study focuses on the aphid host *Acyrthosiphon pisum* and its parasitoid wasp *Aphidius ervi*. Host susceptibility to *A. ervi* in *A. pisum* is clonally variable, depending behaviorally on aphid body color ([29] and references therein), and physiologically on host defensive anti-parasitoid bacterial symbionts. In particular, *Hamiltonella defensa* bacteria can kill or stop development of *A. ervi* immature stages, thus acting as a symbiotic form of host resistance (reviewed in [30]; see also [29], [31], [32], [33]). With host defensive bacteria, the *Aphidius*-aphid host system thus comprises three players associated in a tripartite trophic, symbiotic, and parasitic interaction. Here we consider variation in biological traits of the three players at both the transmission (*A. ervi* wasp) and host exploitation stage (*A. ervi* immature forms) of the *A. pisum*-*A. ervi* relation.

Despite being considered a generalist, *A. ervi* also shows substantial specialization ([34]; see also [12], [35], [36]). Its use of several aphid species as hosts indicates intraspecific variation in virulence and host adaptation [12]. Mostly known as the ‘pea aphid’, *A. pisum* is specialized on different host plants with biotypes or host ‘races’ adapted to crop plants, in particular alfalfa (*Medicago sativa*) and red clover (*Trifolium pratense*) ([37] and references therein; [38], [39]). In our recent study [29], we showed that sympatric *A. pisum* on alfalfa and red clover from three distant sites in Québec belonged to distinct genetic clusters that also vary for anti-parasitoid symbionts, as also recently observed in other populations [40]. However, we found no evidence for *A. ervi* specialization to the genetic host races of *A. pisum* based on virulence indices of host selection and host suitability [29].

Behavior is generally plastic to face new environments [41]. In a potential host parasitoid interaction, novelty may imply an unfamiliar wasp for the host, or an unfamiliar host aphid for the wasp. Just as immunity mismatches occur at the step of parasitism, behavioral mismatches must be considered on both sides. The behavior indices (attack delay, ovipositor contacts) of our previous study [29]) may be too limited to reveal subtle variation in wasp ‘preference’ or host defense, where closely related (subspecific) antagonists interact.

We thus consider the hypothesis that if *A. ervi* is under evolutionary pressure from divergent *A. pisum* host-plant races, wasps may express higher behavioral adaptation to aphids that are most similar to the host they developed from (hereafter, native host). This could be manifested at host recognition (visual or olfaction pathways) or at the level of approaching and attacking to break host behavioral defenses (motor tactics pathway). If divergent parasitoid evolution is in progress, *A. ervi* wasps could more spontaneously recognize, or more easily break defenses of their native aphid host, than alternative (novel/unfamiliar) hosts. Reciprocally, a pea aphid may display more efficient risk recognition and aversion when facing wasps originating from closely related hosts, relative to wasps from alternative host biotypes.

A multivariate approach was used to model success and failure of parasitism as a function of host and wasp behaviors and the host ecological traits of plant affiliation and bacterial symbionts. Based on all sources of host variation (clones, host plant, symbionts) and integrating 2-sided compatibility at the behavioral and parasitological steps, profiles of ‘hospitality’ to parasitism (see discussion) of the host were characterized, which we suggest can be used to better define the complex host polymorphism involved in limiting specialization of its natural enemy.

## Materials and Methods

### Design of experiment

The outcome of a controlled encounter between aphid and wasp was examined in a transplant design combining clonal variation of the aphid (including the host plant association), and wasp lineages originating from different aphid biotypes. A total of 20 host x parasite interactions were evaluated for effects of intraspecific variation of the host and the parasitoid. They consisted of 10 aphid clones randomly isolated from two crop plants (alfalfa and clover, 5 clones from each plant), tested in combination with 2 *A. ervi* lineages differing by the aphid host from which they originated (alfalfa *A. pisum*, clover *A. pisum)*.

### Hosts and parasitoids sources

Aphids were collected in early to midsummer in perennial alfalfa and red clover fields at Laval University's experimental farm, St-Augustin-de-Desmaures, Quebec, Canada [29]. Collected aphids were unwinged asexuals often found in association with their young. They exhibited dimorphic color variation (green or pink) but we did not attempt to estimate proportions of each color morph in the field. Sampled aphids were bred clonally in isolation under controlled conditions (18°C, 16L: 8D photoperiod) on the crop plant of origin (red clover, alfalfa), providing a few hundred clones from which ten clones were randomly selected (Table 1). Along with crop plant of origin and color morph, aphids were characterized for symbiotic bacteria with PCR based on 16SrDNA primers [29], [32], [42]. For each experimental clone, we determined the occurrence of the facultative symbionts *Regiella insecticola*, *Hamiltonella defensa*, *Serratia symbiotica* and PAXS.

**Table 1:** Experimental aphid clones classified by source crop plant, aphid color morph and screened symbiotic bacteria.

	Aphid crop plant	
Color morph	Alfalfa	Clover
**Green**	52Ag0, 451Ag0, 716Ag0	90CgU, 210CgT
**Pink**	702Ap0, 717ApT	734Cp0, 8003Cp0, 8005CpU

**Figure 1:**
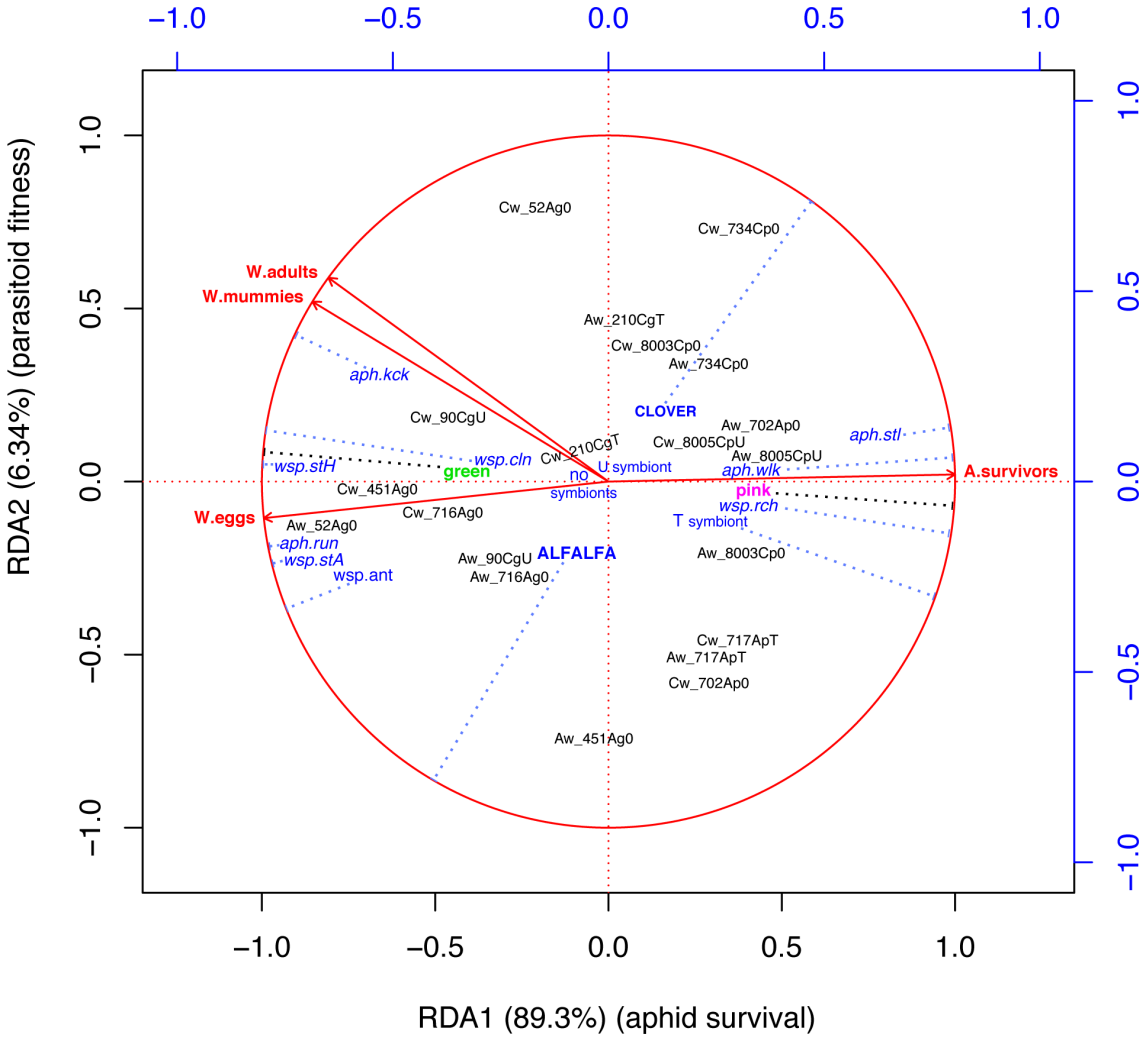
Biplot of RDA on outcomes of encounters of *A. ervi* with potential hosts belonging to 10 clones of two crop plant variants of the aphid *A. pisum*. Responses shown as solid arrowed lines are related to 12 explanatory variables (dotted lines) representing aphid and wasp behaviors, aphid body color, aphid facultative symbiont, and aphid host plant (alfalfa or clover). Projection of variables on unit circle allows measuring the predictive power of explanatory variables on aphid and parasitoid fitness, as their distances on the circle (see text for explanation).

Clone IDs are unique numbers from field samples followed by letters: first capital letter (A or C) is for alfalfa or clover crop; lowercase letter (g or p) is for green or pink; last letter is for aphid symbiont, T for *Hamiltonella defensa*, U for *Regiella insecticola*, or 0 for none of the symbionts tested for (see text for details). In Figure 1, wasp x clone interactions are identified as e.g. interaction ‘Aw_52Ag0’ where an ‘Aw’ *A. ervi* wasp interacts with individuals (n = 6) of the clone ‘52Ag0’.

The experimental *A. ervi* wasp lineages Aw (alfalfa aphid wasp) and Cw (clover aphid wasp) were derived as described in Bilodeau *et al.* [29]. Briefly, field collected parasitized aphids forming ‘mummies’ were reared, and emerging *A. ervi* adult wasps, which could thus be linked to an aphid host whose field crop plant was known, were bred as lab colonies. The experimental wasps were isolated from their respective (Aw and Cw) lineages at the mummy stage, and were mated under direct observation with a male of their colony within 24 h post emergence. At the time of testing, they were aged 48–72 h and (as adult wasps) had no experience with aphids.

### Behavioral observations

Aphid and wasp behaviors were observed in a 5-min encounter at a temperature near 20°C in a small arena (Petri dish 60×15 mm). A midsize (third stage) aphid randomly selected from one of the 10 experimental clones was used, thus controlling for host size effects on wasp and host behavior. The experimental aphid was confined with an *A. ervi* wasp randomly selected from the Aw or the Cw lineages during 5 min, and aphid and wasp behaviors (Table 2) were recorded using The Observer 5.0 software (Noldus Information Technology, Wageningen, Netherlands). All recorded behaviors were overt activities (or inactivity), with more activities from the wasp than the aphid. Walking movements were considered oriented when it was clear that one or the other protagonist directly moved away or toward the other. Aphid first recognition of the wasp as a threat (alertness) was revealed either as freezing (Table 2, *aph.stl* = complete inactivity), slow (non-oriented) movement (*aph.wlk),* or attempted escape (*aph.run)*. Wasp searching behavior *(wsp.mve)* and fortuitous contact (*wsp.cnt)* were assumed to show lack of recognition by the wasp of a potential host present in its close environment. On the other hand, wasp recognition of the aphid as a potential host was revealed as oriented walking toward the host (*wsp.rch)*, and contact with the antennae (*wsp.ant)*, followed by ovipositor attacks to the host body or appendages (*wsp.stH, wspStA)*.

**Table 2:** Explanatory variables examined to predict outcomes of challenges involving 100 *Aphidius ervi* wasps on 600 *Acyrthosiphon pisum* individuals belonging to 10 field-collected clones from either alfalfa or red clover.

Variable	Code in RDA	Remarks
a) Wasp behaviors		
antennal contact	*wsp.ant* (11.09)	palpation of aphid with antennae
non-oriented contact	*wsp.cnt* (1.42)	apparently accidental contact with aphid
resting	*wsp.rst* (13.01)	wasp is completely immobile
oriented walking	*wsp.rch* (40.39)	walking toward aphid or reaching aphid
cleaning	*wsp.cln* (20.64)	cleaning itself with legs and/or mouthparts
ovipositor contact A	*wsp.stA* (6.83)	wasp strikes aphid with ovipositor on abdomen or thorax
ovipositor contact B	*wsp.stH* (1.49)	wasp strikes aphid head or appendages with ovipositor
searching	*wsp.mve* (4.64)	walking rapidly or flying apparently to disperse
b) Aphid behaviors		
inactivity	*aph.stl* (38.58)	aphid is completely immobile
running away	*aph.run* (34.76)	aphid actively walks or runs away from wasp
walking	*aph.wlk* (7.10)	non-oriented move, aphid walks with no directional orientation with respect to the wasp
resistance	*aph.kck* (19.01)	aphid being contacted by wasp actively resists or fights back
cornicle excretion	*aph.exc* (0.55)	aphid excretes fluid from its cornicles (defense)
c) Experimental design effects		
crop plant of aphid	*crop*	alfalfa or clover
host aphid	*host*	alfalfa or clover aphid
crop x host	*crop x host*	interaction
d) Aphid clone-specific variables		
color morph	*color*	green or pink
*Hamiltonella defensa*	*T symbiont*	anti-parasitoid role
*Regiella insecticola*	*U symbiont*	host plant facilitation

Behaviors are listed with their% occurrence recorded for both wasps (*wsp.*) and aphids (*aph.*). Behaviors *wsp.mve* and *aph.exc* were excluded to take into account colinearity imposed by observation time being limited to 5 min. See text for details.

Encounters of wasp individuals with aphids of each clone were replicated on six aphids (N = 6 aphids, the maximum that could be handled), to take into account possible short-term variation in behavior of inexperienced wasps [43], [44], [45], [46]. Replication insured that possible change due to early experience was controlled for in statistical analysis. Immediately after testing a female wasp, a wasp from the other lineage was tested with six new aphids of the same clone. Five complete blocks of this experiment (5 blocks in time) were obtained by having each aphid clone (n = 10) tested on five different wasps from each lineage (N = 5 individuals of each wasp lineage). We thus tested 100 wasps (50 Aw and 50 Cw wasps), in a total of 600 encounters with aphids from the 10 clones that were characterized for color morph, crop plant, and facultative symbionts.

### Host and parasitoid fitness

Following the 5 min test, aphids that were contacted by the wasp and were thus potentially parasitized were partitioned as follows. Replicate aphids #1–3 (n = 300) were dissected under a stereomicroscope within 24 h to determine the number of eggs laid by the wasp. Replicates #4–6 (n = 300) were reared on foliage of their respective crop plant (18°C, 16L: 8D) to observe parasite progeny survival and development up to host mummification and successful adult wasp emergence.

Outcomes of experimental encounters over replicate aphids were used to determine respective fitness of host and parasitoid. Host fitness was measured as a binary variable (*A.survivors)* with values 1 (aphid escaped parasitism in its 5-min close encounter with a wasp) and 0, (aphid failure to escape successful parasitism). Aphid survival (*A.survivors*  = 1) was recorded when i) no parasitoid strikes on the aphid were observed in the 5 min test; or ii) when strikes did occur but failed to result in parasitism as shown by absence of eggs in struck hosts based on host dissection (aphid replicates #1–3); or when normal development and absence of aphid mummification was observed for up to 8 days following the encounter (aphid replicates #4–6). The opposite outcome (successful parasitism, *A.survivors*  = 0), was recorded when the host was attacked and struck, and where eggs were found at dissection, or when struck hosts eventually mummified.

Parasitoid fitness was estimated by the number of eggs and by frequency of parasitoid larvae and adults developed from struck hosts. Three parasitoid fitness variables were estimated, allowing separation of fitness of the adult wasp, from the fitness of its progeny as parasitoids of a host clone. The number of eggs laid in aphid reps #1–3, (*W.eggs)* measures recognition and successful attack of the host (behavioral virulence) by the female wasp during encounters. Wasp fitness variables *W.mummies* and *W.adults* (aphid reps #4–6) estimate the quality of the host for the wasp's progeny (host exploitation virulence), based on number of mummies forming and adults emerging from struck aphids.

### Data analysis

For each combination of aphid clone, wasp lineage, and blocks in time (n = 10×2×5 = 100 combinations), the mean time spent in observed behaviors was calculated over the six encounters of each wasp with a host clone (six aphid replicates). Aphid and parasitoid fitness variables were averaged over replicates used to score them (see above).

We analyzed variation in aphid and parasitoid fitness responses using Redundancy Analysis (RDA) [47], [48]. RDA extends multiple regression to the case of multivariate responses (p. 579 in [47]), as needed here to model the fitness data table (four variables, *A.survivors, W.eggs, W.mummies, W.adults)*. RDA is related to Principal Component Analysis, but differs in that it partitions variables asymmetrically [49] as responses (**Y** matrix of fitness variables) and predictors or explanatory variables (**X** matrix of independent variables). These variables (**X**) were the observed wasp and aphid behaviors and possible effects of controlling the wasp lineage, and the host aphid clone-dependent variables. These were the aphid crop plant, color morph (pink, green) and facultative symbionts (*Hamiltonella defensa* aka T symbiont; *Regiella insecticola* aka U symbiont; or 0 meaning that none of the four facultative symbionts tested for were detected).

Strong colinearity among the explanatory variables was expected, and we first used stepwise regression (backward and forward) to remove any explanatory variables that could be deemed truly insignificant in explaining outcomes, by testing them one at a time. Thus the wasp behaviors *wsp.rst* and *wsp.cnt* (see Table 2), and the design effects parasitoid *Host* and *Host* x *Crop* interaction (both based on controlling the wasp lineage), were eliminated at this step (details not shown). Therefore all other twelve behaviors, and the aphid clone related variables color morph and symbiotic bacteria, as well as experimental factor aphid crop plant (*Crop*) were included in RDA models.

In RDA terms, the objects are the 20 replicated experimental combinations of an individual aphid from an *A. pisum* clone (n = 10) being challenged by an individual wasp from one of the *A. ervi* lineages (n = 2). Each object thus represents a particular host x parasitoid interaction where both players are characterized at the subspecific level, i.e. a wasp lineage (Aw or Cw) with a host clone (ten clones, Table 1). Since each of the 20 interactions (objects) was replicated 30 times (6 aphid individuals×5 wasps), the RDA is based on 600 observations.

RDA was conducted using the function *rda()* in the *vegan* package of R version 2.13.0 (R Development core Team, 2011). We present RDA results based both on sequential modeling effects (significance based on Type 1 error in ANOVA) and marginal effects (ANOVA Type 3 effects), in order to compare and better assess how each explanatory variable contributed to outcomes. Results were interpreted using correlation biplots (scaling 2), where Euclidean distance between interactions (objects) is not preserved, to reveal possible direct relations between response and predictor variables (p. 587 in [47]; [49]). Univariate and multivariate regression models used to complete the RDA allowing us to evaluate the relative contributions of each explanatory variable to the **Y** outcomes variables, and the extent of colinearity among potential explanatory variables.

## Results

*Aphidius ervi* wasps were generally active and responsive to aphids in encounters with individuals of all clones. A large majority of the wasps completed the test over the six aphid replicates within less than 1 h, providing nearly 15,000 observations of aphid and wasp behaviors recorded for occurrence and duration to be analyzed with RDA.

Observed behaviors and host/parasite fitness responses varied across the clone x wasp interactions examined and so did outcomes as summarized here. Mean number of strikes (ovipositor contacts) ranged from 2.00 to 19.20 (n = 20) per aphid, resulting in 0 to 2.00 eggs laid per aphid on average. The ratio of aphid clone acceptance for oviposition (proportion of attacked aphids resulting in parasitism) by *A. ervi* wasps averaged 0 to 0.95, this high value being observed for an alfalfa aphid *A. ervi* wasp facing a pink alfalfa aphid (clone 717ApT). In all clone x wasp interactions (n = 20), the aphids being contacted ≥1 time with the ovipositor produced mummies and adult wasps, with an average emergence ratio (proportion of emerged adults from mummies) ranging from 0.07 to 0.67, and an average female sex ratio ranging 0.0 to 1.0, indicating wide ranging parasite-host compatibility at both the behavioral and host exploitation levels.

Successful RDAs were obtained where twelve **X** variables were retained to explain a large fraction of the variance of the **Y** outcomes i.e. host/parasitoid fitness (Table 3). The first two axes were highly significant, RDA1 (F_1 15_  = 364.026, P≤0.001) capturing 89.3% of outcomes variance, and RDA2 (F_1 15_  = 25.828, P = ≤0.001), an additional 6.34%, totaling 95.6%. Figure 1 shows that variance in aphid and parasitoid fitness is strongly represented by the first axis, with aphid survival and parasitoid fitness indices on opposite sides of the 2D reduced space, defined by the first two RDA axes. RDA1 thus strongly represents both the success (on left side), and the failure of parasitism (right side). Aphid survival (*A.survival)* points directly to the right, almost parallel with RDA1, while all three parasitoid fitness variables point to the left (Fig. 1).

**Table 3:** RDA models testing twelve explanatory variables (see Table 2 and text for details) as predictors of outcomes in encounters between 100 *Aphidius ervi*, with 600 *Acyrthosiphon pisum* individuals from ten clones.

Source	Df	Variance	F	Pr(>F) 1
RDA1	1	0.39750	364.026	≤0.001 ***
RDA2	1	0.02820	25.828	≤0.001 ***
RDA3	1	0.00240	2.195	0.122 ns
RDA4	1	0.00060	0.5502	0.545 ns
Residuals	15	0.01638		
Univariate RDA models testing explanatory variables in isolation				
wsp_rch	1	0.2870	1.3914	0.227 ns
wsp_ant	1	0.6878	3.7376	0.038 *
wsp_stA	1	1.8005	14.7340	0.001 ***
wsp_stH	1	1.3857	9.5412	0.004 **
wsp_cln	1	0.1482	0.6927	0.483 ns
aph_stl	1	0.99475	5.9581	0.007 **
aph_kck	1	0.95768	5.6661	0.012 *
aph_run	1	1.6050	12.062	0.001 ***
aph_wlk	1	0.2547	1.2239	0.278 ns
color morph	1	2.1488	20.894	0.001 ***
symbiont	2	0.3037	0.6985	0.533 ns
crop plant	1	0.1238	0.575	0.535 ns
Residuals	18			
Stepwise regression RDA showing marginal effects (Type 3)				
wsp_rch	1	0.022712	8.3197	0.014 *
wsp_ant	1	0.010737	3.9332	0.068 ns
wsp_stA	1	0.027485	10.0685	0.015 *
wsp_stH	1	0.016420	6.0148	0.030 *
wsp_cln	1	0.030422	11.1441	0.010 **
aph_stl	1	0.023906	8.7571	0.009 **
aph_kck	1	0.031654	11.5953	0.005 **
aph_run	1	0.028431	10.4149	0.007 **
aph_wlk	1	0.015780	5.7806	0.026 *
color morph	1	0.002227	0.8156	0.377 ns
symbiont	2	0.032905	6.0268	0.020 *
crop plant	1	0.025203	9.2324	0.009 **
Residuals	6	0.016379		

1 Results based on non-parametric F and P values (1000 permutations). Signif. codes: 0 ‘***’ 0.001 ‘**’ 0.01 ‘*’ 0.05≥0.05^ ‘ns’^

RDA axes 1 and 2 are highly significant predictors of outcomes. Note discrepancy in significance for several explanatory variables when tested in isolation vs. as marginal effects, in particular aphid color morph.

The second significant RDA2 axis captures a separation between the host recognition and attack response of the wasp (slightly below the origin), from the success of parasitism at the host exploitation level (above the origin) (Fig. 1). The location of response variable number of eggs laid (*W.eggs*) indicates that hosts from interactions (objects) below the origin were attractive and susceptible to wasp challenges, while orientation of fitness variables numbers of parasitoid mummies (*W.mummies)* and emerged adults (*W.adults)* can be used to indicate those choices made by wasps that were the best for their progeny.

**Figure 2:**
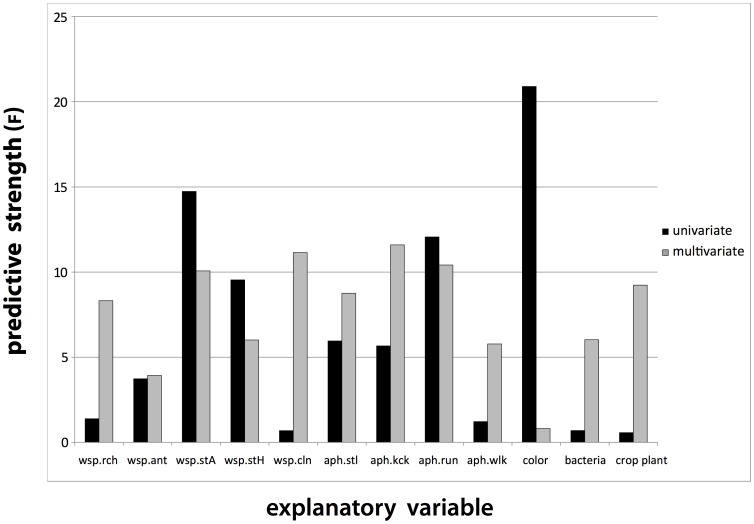
Predictive power of explanatory variables used in RDA models of parasitism of aphid *Acyrthosiphon pisum* by *Aphidius ervi*. Variable aphid color appears as best predictor when tested alone in univariate RDA, but is poorly predictive in multivariate model because of colinearity.

Comparing univariate and multivariate RDA modeling results (Table 3, Fig. 2) reveals important differences. Explanatory variables that appeared highly significant predictors in isolation become insignificant in the multivariate model as marginal effects after adjusting for other explanatory variables. For example the variable aphid color morph is highly significant and the best predictor when treated as the sole univariate predictor (F_1,18_  = 20.89, P≤0.001) but has no significant effect (F_1, 6_  = 0.8156, P = 0.377) in multiple regression modeling. The reverse is observed for aphid crop plant and aphid facultative symbionts, which are insignificant univariate predictors, but are significant effects in multivariate modeling (Table 3). Moreover, among nine behaviors included in the RDA, three insignificant univariate predictors (*wsp.rch, wsp.cln, aph.wlk*) are significant multivariate predictors, the opposite being true for one behavior (*wsp.ant*), which reversed to insignificant.

Positions of the twenty interactions (objects) in the 2D RDA space (Fig. 1) illustrates how each one scored in terms of outcomes, which is obtained by projecting them at a right angle on response variables. Interactions on the right hand side reflect conditions for aphid survival, and conversely for those on the left hand side. We thus see that most but not all of the pink aphid clones appear on the host survival side and, conversely, that most of the green clones are on the parasitoid success side (Fig. 1). This is corroborated by the fitted trend of the color morph variable (green, pink), which is closely parallel to RDA axis 1.

RDA2 explains less variance, but its equally high significance (P≤0.001, Table 3) indicates that positions of interactions on the lower versus the upper-left hand side of the biplot reflect conditions for success of parasitism dependence either on the *A. ervi* wasp recognizing and attacking hosts, versus depending on its progeny efficiently establishing and exploiting the aphids as hosts. We thus see that most aphid clones originating from the Alfalfa crop are more strongly attacked by the wasps, while most clones from the Clover crop are the best hosts for immature parasitoids to complete full development and emergence as adults.

The RDA biplot (Fig. 1) also reveals correlations (or corresponding regression coefficients) between intraset and interset associations of the responses (solid arrowed lines) and the explanatory variables (dotted lines ending as a T on the unitary circle). Correlation is revealed as the angle between lines representing two variables (p. 587 in [47]). High correlation between pairs of variables is clearest when the angle between them tends to 0° (positive correlation) or tends to 180° (negative correlation) (p. 263 in [48]). In Figure 1, correlations between variables are also revealed as the distance between their projections on the unitary circle (dash ending projections). Close proximity of two variables on the circle means high positive correlation, while maximum distance between them in opposed sectors of the circle means high negative correlation. Thus it is possible to identify individual variables or groups of variables having the strongest influence on the outcomes of parasitism as follows.

First with respect to variables related to aphid surviving encounters with wasps, i.e. behaviors that are correlated with response *A.survivors,* we can see that non-oriented walking of both players *(wsp.rch, aph.wlk)* and aphid complete immobility (*aph.stl*) are important behaviors. They may be interpreted as lack of mutual attention, or lack of reaction to each other, where the wasp does not recognize a host as such or ignores it, and where the host either freezes in front of a potential attack, or is slowly moving. We also see that non-behavioral host variables pink color morph, and housing a T symbiont (anti-parasitoid bacterial symbiont *H. defensa*), are closely associated with aphid survival as expected. To a lesser extent aphid survival is also linked with the clover plant association of the aphids (Fig. 1).

Turning to outcomes of parasitism success, and starting with wasp fitness index number of eggs laid (*W.eggs)*, the most strongly correlated wasp behaviors were frequency of direct attacks or ovipositor strikes (*wsp.stA, wsp.stH),* which was expected. They are followed in importance by wasp antennation of the aphid *(wsp.ant),* and self-cleaning activity of the wasp *(wsp.cln)*. Surprisingly, host kicking *(aph.kck)* and running *(aph.run)* behaviors, which were the aphid's most overt defensive behaviors, are also highly correlated to wasp fitness, and thus were evidently inefficient at protecting the aphids in this context. It is also possible that they strongly stimulate wasp attacks. A small angle between wasp cleaning *(wsp.cln)* and aphid kicking (*aph.kck)* (Fig. 1) indicates positive correlation between aphid retaliation with leg kicks and wasp investment in cleaning.

Parasitoid fitness indices number of mummies (*W.mummies)* and adults emerged *(W.adults)* are strongly correlated, showing that mummies generally emerged as adults. Interestingly, kicking behavior of the aphid (*aph.kck)* is the most closely correlated with these response variables (Fig. 1). The non-behavioral explanatory variable green aphid is strongly associated with successful parasitism, correlating about equally to number of eggs laid (*W.eggs)* on the one hand, and numbers of mummies formed (*W.mummies)* and adult wasps emerged (*W.adults)* parasitoid on the other (Fig. 1).

The non-behavioral variable alfalfa crop plant of the aphid correlates with parasitoid fitness, but not strongly and only in terms of fitness index number of eggs laid (*N.eggs*). The Alfalfa - Clover trend of aphid affiliation to crops (Fig. 1) crosses quadrants 1 and 3, such that its relation with parasitism is not simple. However, it is clear that *A. ervi* wasps of both *A. pisum* host origins are found on both sides of RDA1, confirming no influence of their host (alfalfa or clover aphid) on outcomes of parasitism in this experiment. Additionally, the interaction of aphid clones with a given wasp lineage (Aw or Cw) is generally close to its interaction with the other wasp lineage along RDA axis 1, which indicates that aphids from a given clone were nearly equally good or bad host to wasps of both lineages. A possible exception is alfalfa aphid clone 451, which was a high quality host (high fitness) to Cw wasps, but survived very well encounters with the Aw wasps. This also applies, but to a lesser extent, to aphid clones 90 from clover and 52 from alfalfa.

## Discussion

Several studies have previously considered host variation affecting specificity, or lack thereof, of *Aphidius* parasitoids at the inter-specific ([28] and references therein; [12], [50]) and intra-specific levels [9], [51], [52]. The objective here was to study specialization at the intraspecific level (polymorphism), which we did with ten phenotypically well-characterized clones of the host *A. pisum,* and two lineages of the *A. ervi* wasp in a transplant experiment.

Expectations were on the parasitoid behavioral side that if specialized *A. pisum* from clover and alfalfa select *A. ervi* to differentially optimize responses towards their hosts, non-native hosts could not be as recognizable or as stimulating on close contact; or wasp attacks on them should not be as effective. Behavioral differences (genetic, or acquired in host contact during late development) between the experimental *A. ervi* lineages could be associated to their original native host being alfalfa or red clover *A. pisum.* However, this hypothesis clearly is not supported by the data. Experimental factor ‘wasp lineage’ and its interaction with aphid crop as potential effects were both rejected early in the selection of explanatory variables for RDA. Therefore, the more detailed behavioral data and deeper multivariate analyses confirm previous finding that *A. ervi* has not diverged in response to the host plant specialization of its host *A. pisum* ([29]; see also [53]). There may be various explanations for lack of *A. ervi* specialization toward host races of *A. pisum* [29]. An explanation may be that selection on the parasitoid adults versus immature stages is complex and inconsistent, especially due to variation in clonal resistance to parasitism in *A. pisum* being only loosely related to host plant affiliation.

The broad variation in outcomes of the 20 interactions tested in this study can be explained by *A. pisum* clonal variation, which includes crop plant-related variation, but also facultative symbionts, color morphs, as well as correlated aphid behavior patterns expressed in encounters with wasps, as discussed below. Aphids are known to defend behaviorally from parasitoid wasps [54], [55], [56], [57]. That aphid behavioral defenses may vary discretely at the subspecific level (aphid clone or higher level, e.g. host plant biotype), particularly in concert with other aphid traits playing a role in parasitism such as color and anti-parasitoid bacterial symbionts has not received much attention ([58] and references therein).

Our experimental work and RDA analysis results reveal a ‘hospitality’ trend among the *A. pisum* clones tested as hosts, which is well represented in the 2D-reduced ordination (Fig. 1). The concept of ‘hospitality’ of a host to parasites that we proposed here has not previously been used in parasitoid biology, nor has it been applied to parasitic eukaryotes in general. ‘Hospitality’ of an *A. pisum* host entails both intrinsic and extrinsic phenotypic traits (morphological, ecological, symbiotic) making it more or less vulnerable or resistant to parasitism as a whole. The ‘hospitality’ traits or syndrome of *A. pisum* can be delineated (at least in part for some key traits) in interactions with *A. ervi,* based on the status as probable ‘survivors’ or probable ‘losers’ of the *A. pisum* clones in reduced ordination space, using their positions in relation to clusters of explanatory variables on the RDA biplot (Fig. 1).

The ‘inhospitable’ host aphid, a frequent survivor to parasitoids, can be described as being (often but not exclusively) a pink morph exhibiting a tendency to freeze or to move quietly in the presence of a wasp, and which houses a T symbiont. Both *H. defensa*-symbiosis and pink color previously have been found to be linked independently to anti-parasitoid defense in *A. pisum*, we show here how they jointly contribute to the host aphid phenotype as the object of host selection, to favor escape of parasitism. This is well supported by the contrasting explanatory value of these variables when examined as univariate vs. multivariate predictors of parasitism by *A. ervi* (Fig. 2), the case of the pink-green color morph dichotomy being a clear example. We conclude that *A. pisum’*s color is a major phenotypic trait involved in parasitism by *Aphidius* wasps, in accordance with previous studies ([59]; but see also [51]), but it cannot be considered as a uniquely sufficient determinant, and must be considered in the broader context of the ‘hospitality’ syndrome. Note that because the ‘host hospitality’ syndrome explicitly incorporates host morphological and ecological (aphid host plant, bacterial symbionts) phenotypic traits, it extends the idea of behavioral syndromes as currently understood in ecology and evolution (reviewed in [60]).

On the other hand, the loser or ‘hospitable’ *A. pisum* phenotype was most often an aphid from a green clone whose symbiont phenotype excluded *H. defensa*, and responding to wasp presence by running (*aph.run),* and on close contact by leg jerking (*aph.kck*), behaviors which contributed (were correlated to) the most to aphid failure to escape parasitism (Fig. 1). This indicates that aphids that were the most strongly reacting to a wasp's pursuits were also the best supporters of its progeny, having failed to escape its attacks. Wasps may use aphid overt alertness and hyperactivity to recognize potential aphid hosts that are suitable, among other signals triggering attack. This would imply that presumably ‘defensive’ aphid behaviors do not systematically protect them from becoming hosts to these specialized parasitoid wasps, and may even excite them into attacks. Given the context of these experiments, we do not however conclude that these behaviors are defensively useless in nature.

**Figure 3:**
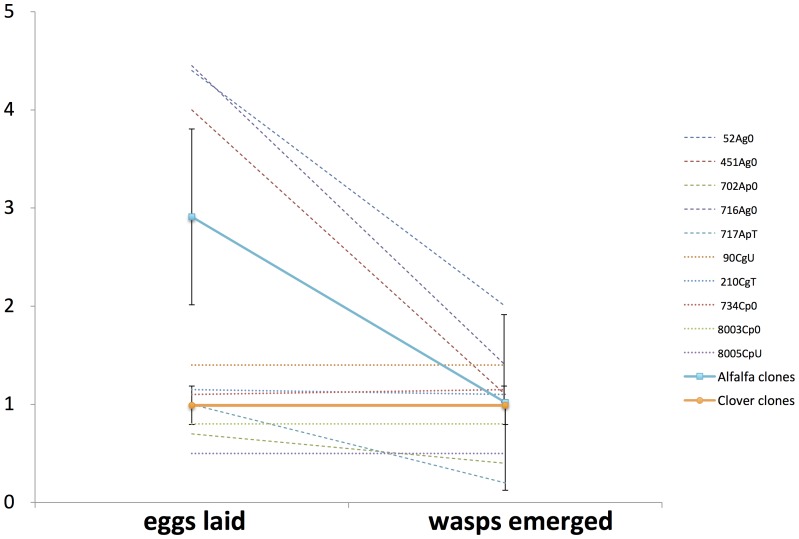
Different fitness trends of infecting (adult female wasp) versus exploiting (egg, larva) stages of *Aphidius ervi* on host aphid clones from alfalfa and clover. Adult females on average laid three eggs on alfalfa clones to produce one adult progeny, compared to one egg on clover aphid clones. The wasp progeny as freshly laid eggs are three times more likely to survive to adult emergence on clover than alfalfa hosts, indicating wasp behavioral compensation at the time of egg laying. Lines added for clarity, there is no implication of linearity in the relationships. Dotted lines are for individual clones (see Table 1), and solid lines with standard error bars represent mean trends (n = 5 clones per crop plant). Letter A or C in clone name stands for the *Acyrthosiphon pisum* subspecies alfalfa or clover.

The aphid host plant, a hypothetically important factor, has an ambiguous role in the ‘hospitality’ syndrome of *A. pisum*. That aphid crop plant was a significant predictor of outcomes in the multivariate RDA (Table 3, P = 0.009; Fig. 2) corroborates its strong structural role of *A. pisum* host populations. However, the distribution of the alfalfa and clover aphid clones with respect to parasitism outcomes in reduced RDA space forms a complex pattern (Fig. 1). Although there were a few exceptions, clones from a given crop were generally either good or bad hosts to *A. ervi*. Overall, clones from clover appeared less hospitable than alfalfa clones, but at the same time clover clones appeared more hospitable to parasitoid exploitation than to recognition as good hosts to *A. ervi* adults. Bilodeau *et al.* [29] had already pointed out an apparent contrast of the relative values of the alfalfa vs. clover *A. pisum* biotypes (or races), as hosts to match the wasp behavioral adaptations (host selection), versus matching the needs of the parasitic stages (host exploitation) (Fig. 3, see also [61]).

Behavioral data support a fitness tradeoff between the two life forms (immature larva, wasp) of *A. ervi* (Fig. 3), where equal fitness on different host races (alfalfa and clover *A. pisum*) depends more on wasp fecundity and egg laying behavior, than immature virulence traits variation. This is supported by regressions of mean number of emerged adults (data from reps #4–6) on mean eggs invested (reps #1–3), where the slope is marginally significant for alfalfa aphid clones (F_1, 3_ =  8.7419, P = 0.060), but clearly insignificant for clover clones (F_1, 3_ =  2.50, P = 0.2021). A fecundity - progeny survival fitness tradeoff for *A. ervi* is further supported by significantly divergent regression of number of adults produced on number of oviposition attacks (alfalfa clones F_1, 3_ = 10.85, P = 0.05; clover clones F_1, 3_ = 3.894, P = 0.143), with an average of 9.76 attacks directed to alfalfa clone aphids, compared to 5.84 on clover aphids. The number of clones tested may be limiting statistical power here, as five clones per crop plant was the maximum that could be handled technically. Results nevertheless support different *A. ervi* fitness trends of the adult phenotype (attack and oviposition behavior) vs. the immature phenotype (resistance breaking, parasitically exploiting the host), on *A. pisum* host biotypes.

Discrepancy between aphid hospitality to the *A. ervi* wasp vs. hospitality to its progeny, where extra eggs are invested to balance immature mortality, is an important finding that emerges from this study (see also [33] who independently observed the same mechanism). In order to exploit a ‘multifaceted’ polymorphic and clonally reproducing host such as *A. pisum*, the *A. ervi* wasps and immatures must balance their differential capacity to use hosts with divergent hospitality occurring together in mixed host populations. This balance should play a role in maintaining the generalist nature of this parasitoid introduced as a biological control agent in North America. Selection by the host on the parasite is mediated in the course of a single generation through sequentially variable host hospitality effects on the adult wasp (differential egg laying), in alternation with host effects on the immature parasitoid (differential survival), which could limit host specialization of *A. ervi* on aphid biotypes associated to different host plants. Coevolving systems are complex and dynamic evolutionary units (p. 492 in [20]). Different fitness gradients of *A. ervi* on two host aphid plant races as observed (Fig. 3) indicates that obligate alternation between its two divergent phenotypes (free-living-wasp, endo-parasitic-larva) has ‘complicating’ consequences that are inherently unfavorable to local adaptation [19].

Populations of *A. pisum* are genetically structured according to host plant [29], but even small populations exhibit discontinuous traits affecting parasitism, in particular anti-parasitoid bacterial symbionts that can modify traits such as aphid body color [62] and behavior [58]. Symbionts are not intrinsic to aphid genetic variation, but aphid hospitality to endosymbiotic bacteria may be a key aspect of aphid genetic variation that deserves further study. Widespread species such as *H. defensa* that vary clonally in aphid populations and as showed previously with aphid host plant biotype [29], [63] cannot be excluded from the aphid phenotype in studying parasitism induced mortality and evolution.
